# A Standardized Maneuver Pattern Library and Dual-View Framework for Multi-View Maneuver Classification

**DOI:** 10.3390/s26051526

**Published:** 2026-02-28

**Authors:** Zhenwei Yang, Zhuang Chen, Botian Sun, Yibo Ai, Weidong Zhang

**Affiliations:** National Center for Materials Service Safety (NCMS), University of Science and Technology Beijing, Beijing 100083, China; d202110534@xs.ustb.edu.cn (Z.Y.); 18813179864@163.com (Z.C.); m202321284@xs.ustb.edu.cn (B.S.); ybai@ustb.edu.cn (Y.A.)

**Keywords:** maneuver pattern classification, Siamese network, dual-view fusion, lightweight architecture, aerial trajectory analysis

## Abstract

Maneuver pattern classification is fundamental for understanding and predicting the dynamic behaviors of aerial vehicles operating in increasingly complex airspace environments. However, existing rule-based and data-driven approaches are constrained by the scarcity, imbalance, and limited maneuver diversity of real-world flight data, leading to a restricted generalization capability and a reduced robustness to noise. To address these challenges, we construct a standardized Maneuver Pattern Library, a curated dataset of simulated flight trajectories encompassing five representative maneuver primitives: climb, descent, left turn, right turn, and loiter. Trajectories are generated using the X-Plane 12 flight simulator under controlled conditions to ensure maneuver diversity and label consistency, refined through noise reduction and cubic spline interpolation, and rendered from synchronized top and side views with time-encoded color gradients to preserve temporal continuity. Building upon this dataset, we propose DualView-LiteNet, a lightweight Siamese convolutional network designed to jointly learn complementary spatial and temporal cues from dual-view trajectory representations through parameter sharing and feature fusion. In addition to comprehensive comparisons with multiple baseline models on the simulated benchmark, we further evaluate the trained model via direct inference on a real-world ADS-B dataset collected from ADS-B Exchange, without any fine-tuning. The consistent performance observed in this sim-to-real setting demonstrates the practical feasibility and generalization capability of the proposed approach. The experimental results show that DualView-LiteNet achieves an accuracy of 97.64%, with its precision, recall, and F1-score all reaching 0.98 on the benchmark dataset, validating its effectiveness for aerial maneuver pattern classification and establishing a reliable reference for future research.

## 1. Introduction

With the rapid development of urban air traffic and integrated space–air–ground networks, aircraft are moving towards a new stage of greater intelligence and collaboration. At the same time, this places higher demands on the aircraft’s behavior. In order to analyze the motion characteristics of aircraft more accurately, it is particularly necessary to study their maneuver patterns. Recognizing these patterns is the core part of aircraft motion characteristics’ analysis. Common flight maneuvers include straight flight, climb, descent, turn, and loiter. Such recognition technology can improve the control capability of aircraft, help detect air traffic conflicts, and assist aircraft in path planning. In other areas, this technology can also be used to train and evaluate pilots, helping them avoid hidden hazards and incorrect maneuvers in a timely manner. As flight missions become increasingly complex and performance standards become increasingly stringent, research on aircraft maneuver patterns is of great significance [1].

The initial phase of research in this area was characterized by a strong dependence on rule-based systems derived from expert knowledge [2,3,4,5]. These systems operated by matching real-time flight parameters against a predefined rule set within an expert-curated knowledge base to identify action types. Although effective in constrained and simple operational contexts, this approach exhibited a critical limitation: its performance was bottlenecked by the scope of manual knowledge engineering. This inherent constraint renders traditional methods increasingly incapable of addressing the demands of modern, high-maneuverability aircraft and dynamically changing battlefields. Notably, the advent of AI and the broader technological revolution have catalyzed a paradigm shift in the field. Data-driven research methods are now unequivocally mainstream. This change is demonstrated by contemporary methods. For instance, Bayesian models and Support Vector Machines (SVM) provide reliable dynamic classification of flight parameters. More effectively, deep neural networks significantly improve pattern identification accuracy by extracting intricate, nonlinear patterns from enormous volumes of flight data [6,7].

Despite notable progress in data-driven maneuver pattern recognition, current approaches exhibit an excessive reliance on flight data. Typically acquired through radar scanning, such data frequently suffers from quality inconsistencies, sensor noise, and compromised temporal integrity, especially under fast-changing maneuver states. These issues lead to trajectory discontinuities, misaligned sampling rates, and viewpoint-dependent distortions, all of which hinder stable feature learning. Consequently, recognition models trained on these datasets tend to demonstrate suboptimal accuracy and limited generalization capability. Furthermore, the inherent scarcity of flight data—particularly the prohibitive costs associated with acquiring high-value maneuver samples in real-world scenarios—poses additional constraints on algorithmic refinement [8,9,10]. Most existing datasets contain only single-view or sparsely sampled trajectories, lacking the structured multi-view representations needed to capture the full geometric and temporal characteristics of complex aerial maneuvers. While current data-driven methods, including both machine learning and deep learning, continue to face challenges such as insufficient accuracy, data inefficiency, and sensitivity to sampling irregularities, research leveraging neural networks for lightweight, robust maneuver recognition remains relatively underdeveloped. Nevertheless, ongoing advances in artificial intelligence, especially the growing maturity of neural network technologies, offer promising new pathways for advancing maneuver pattern recognition [11].

**Figure 1:**
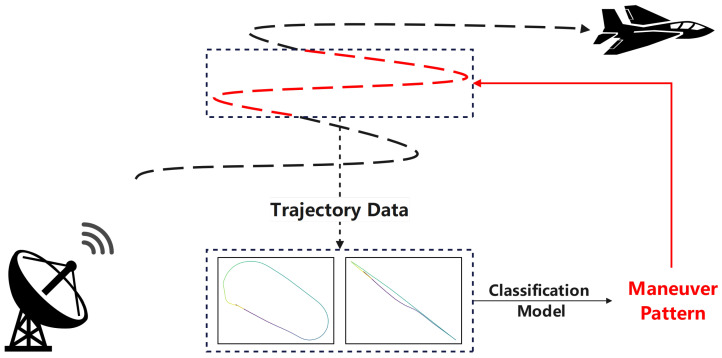
Schematic diagram of maneuver pattern classification. The schematic illustrates the maneuver pattern classification based on top-view and side-view trajectory images. The black dashed curves represent the target’s trajectories, and the red highlighted sections indicate the specific trajectory segments identified for maneuver pattern classification.

To address the long-standing challenges of insufficient model generalization and low recognition accuracy in maneuver pattern recognition, this study integrates data-driven paradigms with deep neural networks to fundamentally optimize the recognition framework. Guided by this core strategy, we first establish a systematic Maneuver Pattern Library as the foundation of our work. Building upon the Maneuver Pattern Library, a dual-view image-based classification approach is proposed, and its overall framework is illustrated in Figure 1. The Maneuver Pattern Library adopts dual-view projection techniques to convert continuous maneuver trajectories into structured images, effectively preserving key temporal features while reducing data dimensionality. This design enhances the representation of trajectory morphology and improves the model’s robustness to data irregularities. In addition, the methodology developed for constructing the Maneuver Pattern Library can be extended to the organization of domain knowledge in aerospace studies, providing useful support for subsequent analyses. To handle the characteristics of dual-view data, a Siamese network is employed, which independently extracts features from each view through convolutional operations and integrates them through a lightweight feature fusion mechanism. Based on this framework, we conduct systematic comparisons with conventional machine learning methods, CNN-based baselines, and attention-enhanced dual-view variants, and further assess the trained model via direct inference on real-world ADS-B trajectories. This study contributes a practical and extensible framework for maneuver pattern recognition and offers a reference for future work in related engineering applications.

The contributions are summarized as follows:

- We construct and release the Maneuver Pattern Library, a structured dual-view, time-encoded image dataset annotated with five fundamental flight maneuver categories.
- We define the dual-view maneuver pattern classification task and demonstrate how orthogonal trajectory projections enhance robustness and interpretability in maneuver pattern classification.
- We establish comprehensive benchmarks for dual-view maneuver pattern classification by comparing conventional machine learning models (SVM), CNN-based approaches, attention-enhanced dual-view baselines, and the proposed lightweight Siamese network DualView-LiteNet. Extensive experiments on simulated data and direct inference on real-world ADS-B trajectories demonstrate the effectiveness and generalization capability of the proposed framework.

The remainder of this paper is organized as follows: Section 2 reviews the related work relevant to this study. Section 3 introduces the construction of the Maneuver Pattern Library. Section 4 presents the proposed DualView-LiteNet framework for the maneuver pattern classification task. Section 5 reports the experimental results and provides detailed performance analysis. Finally, Section 6 concludes the paper and discusses potential future research directions and applications.

## 2. Related Works

### 2.1. Knowledge Base Matching Methods

Early studies on maneuver pattern recognition primarily relied on rule-driven, knowledge-based matching techniques. In these approaches, domain experts predefined a library of canonical maneuver templates that encoded representative flight attributes such as attitude, the angle of attack, and the roll rate. Incoming trajectory segments were then aligned with these templates to infer maneuver categories. Austin [12], for example, formalized pilot knowledge into seven prototypical fighter maneuvers serving as modular components for pattern recognition. Barndt [13] expanded this paradigm by introducing over 1200 expert rules and associating maneuver intensity with structural stress, thereby detecting risky behaviors such as descent pulls and high-G turns. However, such systems often grew cumbersome, with redundant or overlapping logic. To address latency and uncertainty, Tian [14] incorporated fuzzy control theory, smoothing trajectories, labeling key nodes, and filtering perturbations to enhance real-time recognition. Despite their interpretability, knowledge-based frameworks remain limited by their dependence on handcrafted rules and poor scalability in highly dynamic flight scenarios.

### 2.2. Similarity-Based Matching Methods

Similarity-based approaches employ distance metrics or scoring functions to align and compare observed trajectories with standard templates. Dynamic Time Warping (DTW) is the most prevalent technique, automatically stretching or compressing time axes to align key features and compute a similarity score. In the maneuver pattern recognition context, DTW matches a test sequence to prototype maneuvers by minimizing temporal distance, thus handling variations in execution speed [15,16,17,18,19,20,21]. Complementary to DTW, Genetic Algorithms (GAs) have been applied to evolve optimal matching rules without the explicit modeling of maneuver dynamics [22,23,24]. By encoding candidate solutions as chromosomes and applying selection, crossover, and mutation, GAs search the high-dimensional rule space to maximize classification fitness. While similarity-based methods offer interpretability and control, their performance degrades on noisy or incomplete trajectories and they often incur high computational costs.

### 2.3. Machine Learning-Based Methods

With advances in sensing and computing, machine learning techniques such as Bayesian classifiers and SVMs have been adopted to mitigate the limitations of rule-based systems. Bayesian approaches estimate the posterior probability of each maneuver class given features extracted from radar, inertial measurement units, or GPS data—such as velocity, acceleration, the angle of attack, and the roll rate—and assign the class with the highest posterior [25,26,27,28,29,30]. These methods require comprehensive feature sets, yet parameters like the roll rate are not always available in real time, limiting the accuracy and responsiveness. SVMs address small-sample, high-dimensional classification by constructing optimal hyperplanes in feature space via kernel functions [31,32,33,34]. While SVMs generalize well on limited data, they depend on careful feature engineering or time window segmentation to capture temporal dynamics, and their computational complexity can hinder real-time deployment.

### 2.4. Deep Learning-Based Methods

The emergence of deep neural networks has led to end-to-end maneuver pattern classification solutions that learn hierarchical representations directly from raw or minimally processed data. Early work employed autoencoders combined with spectral clustering to reduce dimensionality and segment trajectories [35]. Recurrent neural networks (RNN) [36] have been applied to capture sequential dependencies in flight dynamics. For example, multiple RNN [36] architectures—Bi-LSTM [37], LSTM [38], GRU [39], and vanilla RNN [36]—have demonstrated strong capability in identifying turning and maneuver types of airborne targets [40]. Such temporal models often incorporate sliding windows, dropout, or bidirectional structures to improve stability and robustness in long-range sequence modeling [41,42].

More recently, Transformer architectures—with self-attention mechanisms—have demonstrated their superior ability to model spatiotemporal dependencies, inspiring variants tailored to maneuver classification [43,44,45,46,47,48,49]. However, these architectures typically involve large parameter counts and substantial computational overhead, making them less suitable for lightweight or real-time deployment scenarios. Although a few recent Transformer variants attempt to reduce model complexity through sparse attention, windowed attention, or hierarchical designs, the existing literature still primarily focuses on performance rather than architectural efficiency. In addition to deep neural models, probabilistic graphical models have also been explored. For instance, Markov Random Field (MRF)-based segmentation methods have been proposed to recognize and partition flight actions, achieving a higher accuracy than traditional flight action recognition algorithms [50]. These models provide structured interpretability but generally lack the flexibility and representation power of modern neural networks.

While knowledge-based and similarity-based approaches offer interpretability and controllability, they lack robustness in complex or noisy environments. Traditional machine learning improves generalization but requires extensive feature engineering. Deep learning methods excel in feature extraction and end-to-end learning, yet they are often computationally heavy and data hungry. Existing studies rarely adopt explicitly lightweight architectures for maneuver classification, leaving a gap between high-capacity models and resource-constrained applications. This motivates the development of our lightweight dual-view framework, which aims to maintain competitive recognition performance while significantly reducing computational complexity and dependency on large-scale labeled datasets.

## 3. Maneuver Pattern Library

The Maneuver Pattern Library serves as the dataset for maneuver pattern recognition and constitutes the foundational focus of this research. While previous recognition models often directly analyze time series data, trajectory time series data is susceptible to influences from data sources, with actual trajectory data frequently exhibiting significant fluctuations. To address this, our study leverages projection-based dimensionality reduction to convert trajectory data into image features, thereby constructing a comprehensive Maneuver Pattern Library to support subsequent investigations.

**Figure 2:**
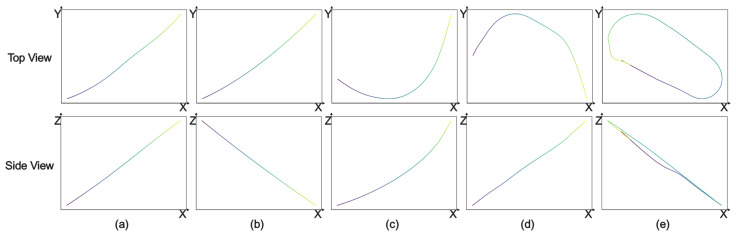
Illustration of the Maneuver Pattern Library. This figure presents a schematic overview of the Maneuver Pattern Library. The top row shows the top-view projections of each maneuver, while the bottom row shows the corresponding side-view projections. Each column represents a specific maneuver type: (**a**) climb maneuver, (**b**) descent maneuver, (**c**) left-turn maneuver, (**d**) right-turn maneuver, and (**e**) loiter maneuver. A color gradient from dark to light indicates the progression from the start to the end of the trajectory.

Figure 2 provides a detailed visualization of the five maneuver categories included in the Maneuver Pattern Library—climb, descent, left turn, right turn, and loiter—each shown in both top-view and side-view projections. A color gradient from dark to light indicates the progression from the start to the end of the trajectory. These complementary views illustrate why single-view observation is often insufficient for reliable maneuver discrimination. For example, climb and descent trajectories appear almost linear in the top view, yet are distinguished in the side view by whether the end point lies above or below the start. Conversely, left turn, right turn, and loiter maneuvers require top-view information: turning direction is inferred from the horizontal curvature and relative positioning of start and end points, while loiter is identifiable by its closed or near-closed loop pattern. By presenting both projections, Figure 2 qualitatively demonstrates the necessity of dual-view fusion and provides intuitive evidence of how different maneuvers manifest in complementary geometry. These visualizations support the construction of a robust Maneuver Pattern Library and directly motivate the dual-view modeling strategy employed in this work.

### 3.1. Data Generation

Since real aircraft flight data are often confidential due to military and commercial restrictions, and may suffer from radar inaccuracies or data loss, this study employs a flight simulation platform to generate high-quality data for subsequent analysis. Mainstream simulation systems include Microsoft Flight Simulator (MFS) [51,52], FlightGear [53], Prepar3D [54], Digital Combat Simulator World (DCS World) [55], and X-Plane 12 [56,57,58,59,60,61,62,63]. MFS, developed by Microsoft since 1982, features ray tracing and photorealistic environments, offering diverse aircraft and weather settings. FlightGear, launched in 1997 as an open-source project, allows users to modify and extend its realistic flight models freely. Prepar3D, developed by Lockheed Martin in 2010, focuses on professional training and supports plugin extensions with real-time meteorological and navigation systems. DCS World, introduced by Eagle Dynamics in 2012, specializes in military flight and air combat simulation, featuring an advanced physics engine for realistic aerial maneuvers. X-Plane 12, released in 2022 by Laminar Research, employs Blade Element Theory to precisely model aerodynamic behavior and integrates modern graphics and dynamic weather systems.

Each platform has unique strengths and limitations. MFS offers superior graphics but demands high hardware performance and is less suitable for extreme maneuvers. Prepar3D provides realistic environments but is costly and complex to use. DCS World focuses mainly on combat scenarios, limiting its general applicability. FlightGear, though open source, lags behind in terms of its graphics and physics realism. X-Plane 12, in contrast, achieves a strong balance between realism, flexibility, and usability. Its physics engine accurately reproduces stall and adverse weather behavior, and its built-in aircraft, airports, and exportable flight data make it ideal for research purposes. Accordingly, X-Plane 12 is selected as the main platform in this study. It provides authentic, controllable, and diverse flight data that effectively support trajectory analysis and maneuver pattern recognition.

**Figure 3:**
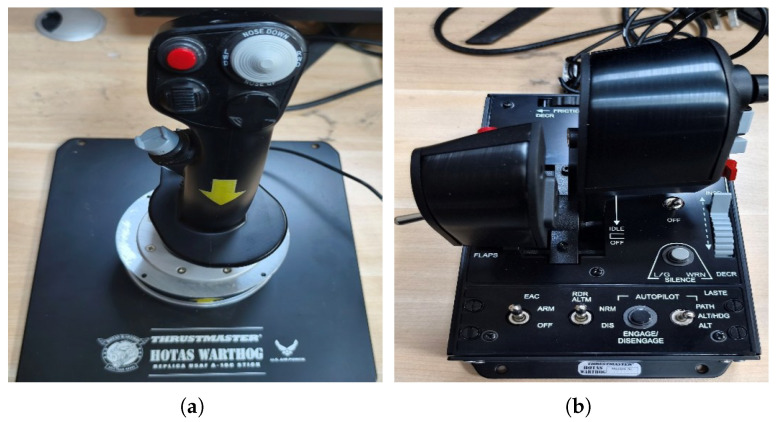
Flight simulation equipment. (**a**) Control stick; (**b**) throttle quadrant.

**Figure 4:**
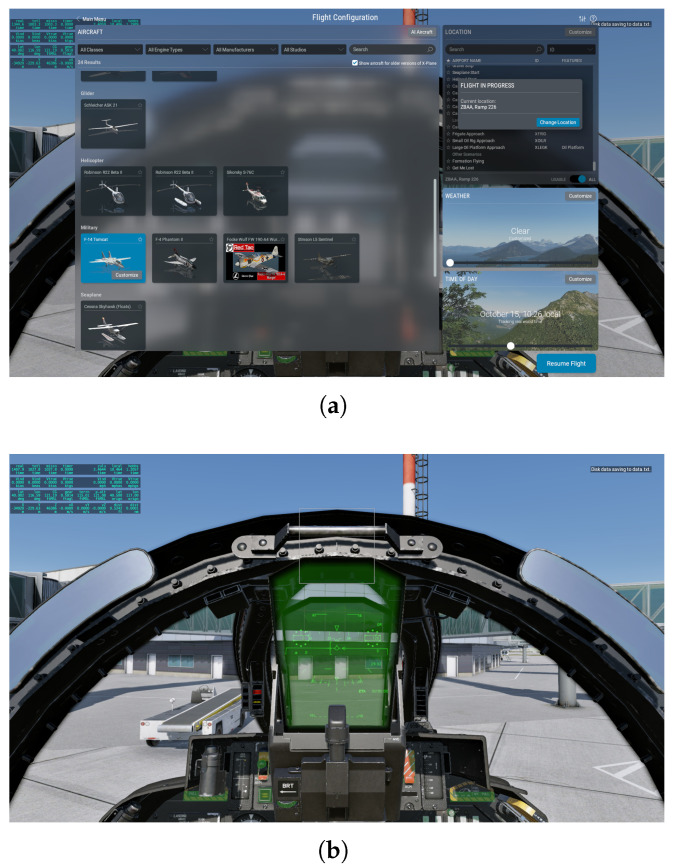
Illustration of the flight page. (**a**) Flight configuration; (**b**) flight interface.

As seen in Figure 3, all simulations use a desktop hardware setup consisting of a Thrustmaster A-10C “Warthog” joystick and throttle system (Guillemot Corporation S.A., Carentoir, France). The joystick’s three-axis magnetic sensors and 19 programmable buttons enable the precise control of roll, pitch, and yaw inputs, while the throttle’s adjustable friction mechanism ensures stable and smooth power transitions during maneuver execution. Leveraging these controls, we pilot a Grumman F-14 “Tomcat” (Grumman Aerospace Corporation, Bethpage, NY, USA) through the prescribed maneuver set, as illustrated in Figure 4. It should be noted that the choice of the F-14 is not motivated by military applications, but rather by its role as a well-established and thoroughly documented aerodynamic platform within the simulation environment. The proposed framework does not depend on aircraft-specific parameters, and the maneuver patterns are defined purely based on trajectory geometry and kinematics. The F-14 is a twin-seat, twin-engine, variable-sweep wing aircraft whose aerodynamic characteristics and structural design have been extensively documented over decades of use. The detailed physical and performance parameters of the aircraft are summarized in Table 1. Its propulsion system originally featured the TF30-P-414A turbofan engines (Pratt & Whitney, East Hartford, CT, USA) and was later upgraded to F110-GE-400 engines (General Electric, Cincinnati, OH, USA), each capable of delivering up to 123 kN of maximum afterburning thrust. This combination of well-established aerodynamic data, stable performance characteristics, and strong maneuverability makes the F-14 a representative and reliable platform for generating consistent and realistic maneuver trajectory data in simulation.

The simulation environment is configured around Runway 11L of Beijing Daxing International Airport, a modern large-scale civil airport with a 3796 m long and 60 m wide runway. Selecting a major international airport provides a standardized and obstacle-free environment, reducing confounding factors from terrain or runway limitations and ensuring that takeoff, landing, and low-altitude procedures remain consistent across repeated runs. Weather conditions are intentionally set to clear skies with 64 km visibility, 58 °F temperature, an altimeter setting of 1013 hPa, and a wave height of 3 ft from direction 270°. These parameters establish a controlled baseline with minimal atmospheric disturbance, allowing maneuver differences to arise primarily from pilot inputs rather than stochastic environmental effects. This controlled setting enhances the reproducibility of trajectories and isolates the effect of maneuver type on trajectory variation. Under this standardized environment, we conducted 14 simulation runs covering the five target maneuver categories, each recorded at high temporal resolution to ensure the fidelity of the resulting 3D trajectory dataset. The detailed process of segmenting these continuous trajectories into discrete maneuver samples, along with the final distribution of samples across the five classes, is described in Section 3.3.

### 3.2. Coordinate Transformation

The raw trajectory data are recorded in WGS84 geographic coordinates (latitude, longitude, and altitude), which are unsuitable for direct Euclidean distance computation. To obtain a unified metric 3D representation, we convert all points into the geocentric Cartesian system CGCS2000, whose origin is at the Earth’s center of mass and whose axes align with the prime meridian (X), the 90° E meridian (Y), and the rotational axis (Z).

For each trajectory point with geodetic latitude ϕ, longitude λ, and altitude *h*, we compute the radius of curvature in the prime vertical as $N=a/\sqrt{1-e^{2}\sin^{2}\phi }$, where a=6,378,137.0 m is the WGS84 equatorial radius and $e^{2}=2f-f^{2}$ is the first eccentricity squared derived from the flattening f=1/298.257222101. The Cartesian coordinates are then obtained by

(1) $$X=\left(N+h\right)\cos\phi \cos\lambda ,\;Y=\left(N+h\right)\cos\phi \sin\lambda ,\;Z=(\left(1-e^{2}\right)N+h)\sin\phi .$$

This conversion produces a consistent Euclidean 3D trajectory representation suitable for subsequent spatial analysis.

### 3.3. Maneuver Pattern Annotation and Data Augmentation

Since no standardized scheme exists for maneuver pattern segmentation, we develop a semi-automated annotation tool in Python (version 3.10.8, Python Software Foundation, Wilmington, DE, USA) using Matplotlib (version 3.10.3, NumFOCUS, Austin, TX, USA) for trajectory visualization and manual labeling. Rather than relying on subjective judgment, the annotation process follows explicit kinematic and geometric rules derived from standard maneuver definitions. Specifically, climb and descent maneuvers are identified based on sustained monotonic changes in the vertical coordinate, with their horizontal projections remaining approximately linear over the corresponding time interval. Left and right turns are determined by the curvature direction of the trajectory in the horizontal plane, where clockwise and counterclockwise bending patterns correspond to right and left turns, respectively. Loiter maneuvers are identified by closed-loop or near-circular patterns in the horizontal trajectory projection, typically spanning multiple revolutions or a continuous looping segment.

Annotators use the visualization tool to inspect trajectories and record the start and end timestamps of maneuver segments according to these predefined rules. The role of the annotators is to apply the criteria consistently and ensure the temporal coherence of maneuver boundaries, rather than to perform subjective or experience-driven interpretation. This rule-based annotation strategy improves reproducibility and enables the systematic construction of a maneuver-centric dataset.

To address class imbalance and enhance robustness, we apply two augmentation strategies:

1. Spatial Translation: Add small random offsets to each Cartesian coordinate, using horizontal displacement ranges of 10 m and 50 m to simulate realistic GPS drift while preserving the maneuver’s global geometric structure.
2. Random Temporal Sampling: Subsample points along the trajectory by adjusting the sampling density to 0.7 or 0.8, mimicking the variability in the onboard measurement frequency without altering the temporal ordering.

Combining these methods yields additional synthetic samples that maintain maneuver characteristics yet diversify spatial and temporal contexts. From the 14 raw trajectories, we annotate and augment to obtain the following sample counts: climb (133), descent (148), left turn (118), right turn (107), and loiter (126), totaling 632 original samples. Post-augmentation, the maneuver library comprises 1264 samples. Although these augmented samples originate from the limited set of raw trajectories, the introduced spatial perturbations and temporal resampling effectively simulate realistic measurement noise and natural intra-class variability—such as slightly steeper or smoother altitude transitions or modest differences in turn curvature. This strengthens the model’s robustness and generalization. However, we acknowledge that augmentation cannot create fundamentally new maneuver types or unseen combinations beyond those embodied in the original flights; thus, the dataset’s intrinsic diversity remains constrained by the scope of the initial raw trajectories.

### 3.4. Trajectory Denoising via Kalman Filter

Although X-Plane 12 provides high-fidelity dynamics, simulation noise remains. We adopt a Kalman filter for recursive “predict correct” smoothing [64]. The state transition is denoted as

(2) $${\bar{x}}_{k}=Ax_{k-1}+Bu_{k}+w_{k},$$

(3) $${\bar{P}}_{k}=AP_{k-1}A^{T}+Q,$$

where ${\bar{x}}_{k}$ is the estimated system state at time step *k*, *A* is the state transition matrix, *B* is the control input matrix, $u_{k}$ is the control input, $w_{k}$ is the process noise, ${\bar{P}}_{k}$ is the state covariance matrix, and *Q* is the process noise covariance matrix.

The update step is formulated as

(4) $$K_{k}={\bar{P}}_{k}H^{T}{\left(H{\bar{P}}_{k}H^{T}+R\right)}^{-1},$$

(5) $$x_{k}={\bar{x}}_{k}+K_{k}\left(z_{k}-H{\bar{x}}_{k}\right),$$

(6) $$P_{k}=\left(1-K_{k}H\right){\bar{P}}_{k},$$

where $K_{k}$ is the Kalman gain, $z_{k}$ is the measurement at step *k*, *H* is the observation matrix, *R* is the measurement noise covariance, and *I* is the identity matrix.

We determine the process noise covariance *Q* and measurement noise covariance *R* through a grid search over the range [0.01,0.05]. For each parameter pair, three quantitative metrics were evaluated: (i) the mean-squared error (MSE) between the filtered and raw trajectories, reflecting fidelity; (ii) the trajectory length, representing the trade-off between smoothness and over-smoothing; and (iii) the acceleration variance (second-order difference variance), indicating motion stability. Sensitivity analysis shows that MSE varies only slightly within Q±0.002, while the acceleration variance is more sensitive to changes in *R*. The parameter combination Q=0.02 and R=0.03 lies in a Pareto-optimal region that balances smoothness and fidelity, and is therefore adopted as the final configuration.

### 3.5. Dual-View Projection and Cubic Spline Interpolation

To reduce dimensionality while retaining maneuver-specific information, each 3D segment is projected onto two orthogonal planes:

- Top view (X-Y) for horizontal turns and loiter loops;
- Side view (X-Z) for climbs, descents, and pitch variations.

Projected points are often sparse, so we employ cubic spline interpolation to reconstruct continuous curves. For ordered points $x_{0},x_{1},\ldots ,x_{n}$, we define piecewise polynomials [65,66]:

(7) $$S_{i}\left(x\right)=a_{i}+b_{i}\left(x-x_{i}\right)+c_{i}{\left(x-x_{i}\right)}^{2}+d_{i}{\left(x-x_{i}\right)}^{3},$$

where $S_{i}\left(x\right)$ is the cubic spline function in the *i*-th interval, $x_{i}$ is the knot point, and $a_{i}$, $b_{i}$, $c_{i}$, and $d_{i}$ are the spline coefficients to be solved.

These splines satisfy continuity constraints on the function values and the first and second derivatives at interval boundaries. Boundary conditions are defined with zero second derivatives at end points to ensure natural spline behavior. Solving the resulting linear system yields smooth, non-oscillatory trajectories suitable for image rendering.

### 3.6. Temporal Color Encoding

After the trajectory data undergoes projection processing, the resulting trajectory lines contain only spatial position information, as the temporal characteristics from the original data are no longer preserved. This makes it difficult for recognition models to extract temporal information. The temporal information of trajectories captures key features such as velocity and acceleration. To address this issue, this study assigns colors along the timeline to the trajectory lines, utilizing gradual variations in color intensity to visually represent temporal evolution. The core concept of color mapping involves assigning colors to trajectory points based on temporal information, creating a visual gradient from dark to light along the trajectory from start to end. We begin by normalizing the timestamp corresponding to each trajectory point:

(8) $$t_{i}^{\prime }=\frac{t_{i}-t_{\min}}{t_{\max}-t_{\min}},$$

where $t_{i}$ is the timestamp of the *i*-th trajectory point, and $t_{\min}$ and $t_{\max}$ are the start and end times of the trajectory, respectively.

Next, the normalized time values are mapped to the hue channel of the HSV color model, causing the trajectory color to evolve over time. In the HSV model, the hue (H, measured in degrees from 0° to 360°) governs the trajectory color, while saturation (S, ranging from zero to one) and value (V, ranging from zero to one) remain fixed to maintain smooth color transitions. The mapping is defined as follows:

(9) $$H_{i}=H_{\min}+\left(H_{\max}-H_{\min}\right)\cdot t_{i}^{\prime },$$

where $H_{\min}$ and $H_{\max}$ define the color gradient range. To prevent abrupt color shifts, linear interpolation is applied to smooth the color transitions, ensuring a visually coherent color evolution. This approach effectively embeds temporal information directly into the 2D trajectory visualization, allowing for the intuitive perception of time-based progression from the start to the end point.

The final library comprises 1264 paired top-down and side-view images, each exhibiting smooth trajectories and clear temporal gradients, as seen in Figure 2. This standardized dataset underpins the Siamese convolutional network recognition framework described in Section 4.

## 4. Method

**Figure 5:**
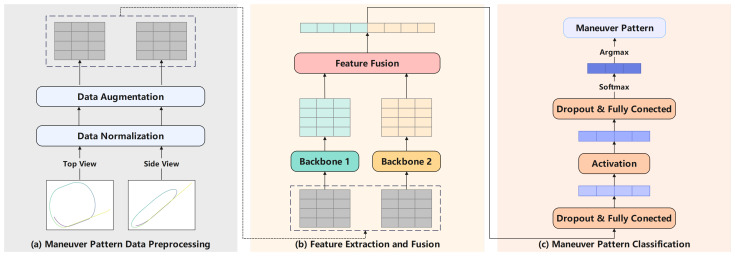
Maneuver pattern classification framework.

This section introduces the DualView-LiteNet framework for maneuver pattern classification, building upon our constructed Maneuver Pattern Library that provides a structured dataset of annotated flight trajectories. As depicted in Figure 5, the framework strategically leverages synchronized top-view and side-view representations from the Maneuver Pattern Library to capture consistent spatiotemporal relationships across diverse maneuvers.

The architecture comprises three core stages: (a) maneuver pattern data preprocessing, (b) multi-view feature extraction and fusion, and (c) maneuver classification. A key design innovation lies in the adoption of a shared-weight Siamese convolutional structure, which enables the learning of unified feature representations from dual-view inputs while maintaining parameter efficiency. By simultaneously processing horizontal (top-view) and vertical (side-view) motion dynamics, the network demonstrates enhanced resilience to perspective variations and significantly improves inter-class discrimination. These deliberate architectural choices allow DualView-LiteNet to achieve an optimal balance between recognition accuracy and computational demands, addressing the critical need for deployable real-time systems in resource-constrained operational environments.

### 4.1. Task Description

The task of maneuver pattern classification based on dual-view trajectory imagery aims to identify the type of aerial maneuver performed by a target through its spatiotemporal motion signatures observed from two complementary perspectives. Each sample consists of a synchronized pair of time series trajectory images: the top view, which represents the planar motion across horizontal coordinates, and the side view, which depicts altitude changes over time. Together, these two projections characterize the aircraft’s motion in both lateral and vertical dimensions, offering a more complete depiction of flight behavior.

Formally, the input to the model is defined as

(10) $$I_{\mathrm{top}},I_{\mathrm{side}}\in R^{H\times W\times 3},$$

where $I_{\mathrm{top}}$ and $I_{\mathrm{side}}$ denote the top-view and side-view trajectory images respectively, each with spatial resolution (H,W) and three RGB channels encoding both spatial contours and temporal gradients.

The corresponding label for each trajectory pair is defined as

(11) y∈{1,2,…,C},

where *C* is the number of maneuver categories, including climb, descent, left turn, right turn, and loiter.

The goal is to learn a mapping function,

(12) $$f:R^{2\times H\times W\times 3}\to \left\{1,2,\ldots ,C\right\},$$

that accurately maps the input image pair $(I_{\mathrm{top}}$ and $I_{\mathrm{side}})$ to its corresponding maneuver pattern label *y*.

### 4.2. Architecture of DualView-LiteNet

DualView-LiteNet is a lightweight Siamese CNN designed for maneuver pattern classification from synchronized top-view and side-view trajectory images. As shown in Figure 5, the architecture comprises three key stages: (a) data preprocessing, (b) dual-branch feature extraction with shared weights and feature fusion, and (c) maneuver pattern classification.

#### 4.2.1. Data Preprocessing

As illustrated in Figure 5a, all raw trajectory images undergo a uniform preprocessing pipeline to ensure consistent inputs and stable model training. First, synchronized top-view and side-view frames corresponding to the same maneuver instance are paired and temporally aligned. Each image is then resized to 224×224 pixels while maintaining the original aspect ratio to fit the network’s expected input size. To mitigate illumination and sensor differences, pixel values are normalized by subtracting the channel-wise mean and dividing by the standard deviation. Finally, the processed images are converted into tensors of shape [3,224,224], compatible with PyTorch (version 2.7.1, Linux Foundation, San Francisco, CA, USA)-style model input conventions.

#### 4.2.2. Dual-Branch Feature Extraction

**Figure 6:**
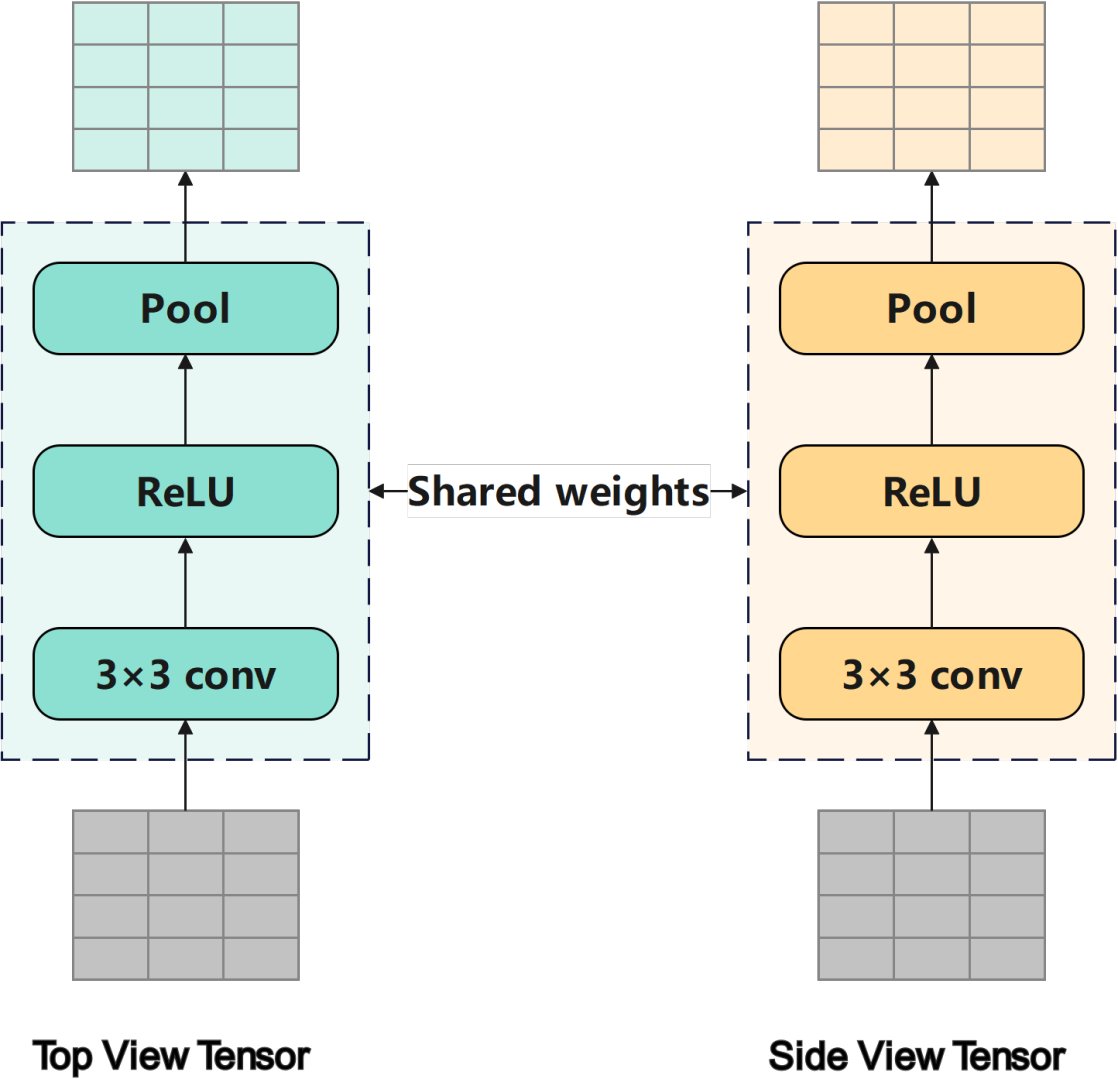
Backbone module. A Siamese dual-branch CNN with shared weights that extracts horizontal trajectory patterns from the top view, and vertical trajectory patterns from the side view.

As shown in Figure 6, corresponding to the Backbone 1 and Backbone 2 components in Figure 5b, the feature extraction module is designed to capture both horizontal motion patterns from the top view and altitude dynamics from the side view. A Siamese convolutional architecture with two identical branches is employed, following a design widely used in metric learning to promote consistent feature representation. At the initialization stage, both branches share identical convolutional weights. During training, the gradients are synchronized across branches, ensuring that parameter updates remain consistent. This shared weight mechanism enables the model to retain view-specific characteristics while benefiting from the mutual reinforcement of the dual branches, effectively improving generalization.

Each branch consists of two consecutive convolutional stages. In each stage, a 3×3 convolutional layer captures local spatial and texture information:

(13) $$F_{ij}^{l}=\sum_{m=0}^{M-1}\sum_{n=0}^{N-1}W_{mn}^{l}X_{\left(i+m)(j+n\right)}^{l-1}+b^{l},$$

where $W_{mn}^{l}$ and $b^{l}$ denote the weights and bias of layer *l*, and $X^{l-1}$ is the input feature map. An ReLU activation function σ(x)=max(0,x) follows to introduce nonlinearity and prevent vanishing gradients, and a 2×2 max pooling operation is then applied to reduce spatial resolution and enhance translational robustness:

(14) $$P_{ij}^{l}=\max_{m,n\in \{0,1\}}F_{\left(2i+m)(2j+n\right)}^{l}.$$

By stacking two such convolution–activation–pooling blocks, each branch can progressively extract higher-level representations of the trajectory structure without incurring unnecessary model depth. This configuration achieves a balance between representational power and parameter efficiency, and subsequent experiments confirm its effectiveness in the maneuver pattern classification task.

#### 4.2.3. Feature Fusion

**Figure 7:**
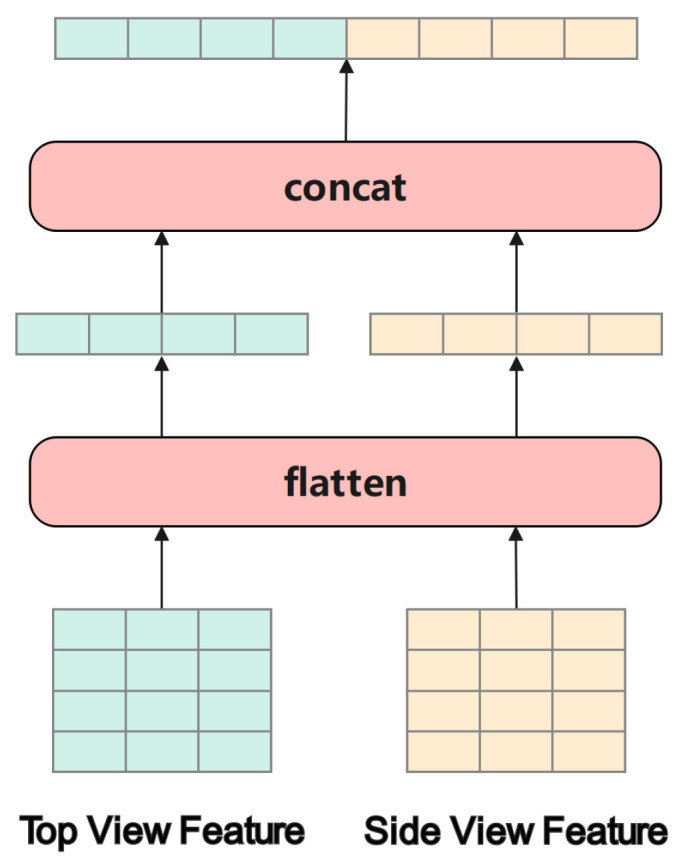
Feature fusion module. Illustration of the flatten–concatenate strategy used to merge top-view and side-view feature maps into a joint representation.

The feature fusion module integrates the outputs of the Siamese network’s top-view and side-view branches by flattening and concatenating their respective feature maps, as depicted in Figure 7 (corresponding to the feature fusion block in Figure 5b). Let $F_{\mathrm{top}}\;$ and $F_{\mathrm{side}}\in R^{H\times W\times C}$ denote the high-dimensional feature maps produced by the two branches, where *H*, *W*, and *C* are the height, width, and number of channels, respectively.

Each feature map is first transformed into a one-dimensional vector [67]:

(15) $$F_{top}^{flat}=\mathrm{Flatten}F_{top},$$

(16) $$F_{side}^{flat}=\mathrm{Flatten}F_{side},$$

where $F_{top}^{flat}\in R^{H\cdot W\cdot C}$ and $F_{side}^{flat}\in R^{H\cdot W\cdot C}$ are the one-dimensional vectors obtained by flattening the original three-dimensional tensors in $R^{H\cdot W\cdot C}$.

These flattened vectors are then concatenated along the channel dimension to form a unified embedding:

(17) $$F_{fusion}=\mathrm{Concat}\left(F_{top}^{flat},F_{side}^{flat}\right)\in R^{2\cdot H\cdot W\cdot C},$$

where $F_{fusion}$ denotes the fused feature vector of dimension $R^{2\cdot H\cdot W\cdot C}$, formed by concatenating the two flattened vectors along the channel axis.

The resulting fused vector $F_{fusion}$ combines complementary spatial features from the top-view branch with altitude dynamics from the side-view branch. This simple yet effective concatenation preserves multi-dimensional information relevant to both horizontal and vertical maneuver patterns, thereby enhancing classification accuracy and robustness.

#### 4.2.4. Maneuver Pattern Classification

The classification head converts the fused feature vector into maneuver pattern predictions through a compact fully connected network followed by a Softmax layer. As shown in Figure 5c, two linear transformations are sequentially applied, each followed by an ReLU activation to introduce nonlinearity and enhance feature discriminability.

Given the fused feature $X\in R^{D}$, the first transformation maps it into a 512-dimensional latent space [68]:

(18) $$H_{1}=\mathrm{ReLU}\left(W_{1}X+b_{1}\right),$$

where $W_{1}\in R^{512\times D}$ and $b_{1}\in R^{512}$ represent the corresponding weights and biases. The intermediate representation is then further compressed to 128 dimensions through another linear ReLU transformation [68]:

(19) $$H_{2}=\mathrm{ReLU}\left(W_{2}H_{1}+b_{2}\right),$$

with $W_{2}\in R^{128\times 512}$ and $b_{2}\in R^{128}$. Finally, the 128-dimensional vector is linearly projected to C=5 logits and normalized via the Softmax function [69]:

(20) $$\hat{y}=\mathrm{Softmax}\left(W_{3}H_{2}+b_{3}\right),$$

where $W_{3}\in R^{C\times 128}$ and $b_{3}\in R^{C}$. The output $\hat{y}\in R^{C}$ provides the probability distribution across the five maneuver categories, and the class with the highest probability is selected as the prediction result.

This two-layer fully connected design efficiently compresses the high-dimensional fused features into a compact representation suitable for classification. By maintaining a simple yet expressive architecture, it achieves a favorable balance between computational efficiency and classification accuracy, making it well-suited for real-time inference scenarios.

### 4.3. Evaluation Metrics and Analysis

To comprehensively assess the performance of DualView-LiteNet on the multi-class maneuver pattern classification task, four widely used evaluation metrics are adopted: overall accuracy, weighted precision, weighted recall, and the weighted F1-score. Together, these indicators capture both global correctness and class-specific behavior, which is particularly important when the data distribution is imbalanced.

The overall accuracy is the fraction of correctly classified samples:

(21) $$\mathrm{Accuracy}=\frac{\sum_{i=1}^{C}{\mathrm{TP}}_{i}}{\sum_{i=1}^{C}\left({\mathrm{TP}}_{i}+{\mathrm{FP}}_{i}+{\mathrm{FN}}_{i}\right)},$$

where *C* is the number of classes, and for class *i*, ${\mathrm{TP}}_{i}$, ${\mathrm{FP}}_{i}$, and ${\mathrm{FN}}_{i}$ denote true positives, false positives, and false negatives, respectively.

The weighted precision is the average of per-class precision values, weighted by the number of true instances in each class:

(22) $${\mathrm{Precision}}_{w}=\sum_{i=1}^{C}\frac{N_{i}}{N}\times \frac{{\mathrm{TP}}_{i}}{{\mathrm{TP}}_{i}+{\mathrm{FP}}_{i}},$$

where $N_{i}$ is the number of ground truth samples in class *i*, and $N=\sum_{i=1}^{C}N_{i}$.

The weighted recall is similarly defined as

(23) $${\mathrm{Recall}}_{w}=\sum_{i=1}^{C}\frac{N_{i}}{N}\times \frac{{\mathrm{TP}}_{i}}{{\mathrm{TP}}_{i}+{\mathrm{FN}}_{i}},$$

where $N_{i}$ is the number of ground truth samples in class *i*, $N=\sum_{i=1}^{C}N_{i}$, and ${\mathrm{FN}}_{i}$ denotes false negatives for class *i*.

The weighted F1-Score is defined as the harmonic mean of the weighted precision and weighted recall, and therefore serves as a summary indicator that jointly reflects the balance between these two complementary aspects of model performance:

(24) $${\mathrm{F1-Score}}_{w}=2\times \frac{{\mathrm{Precision}}_{w}\times {\mathrm{Recall}}_{w}}{{\mathrm{Precision}}_{w}+{\mathrm{Recall}}_{w}},$$

where ${\mathrm{Precision}}_{w}$ and ${\mathrm{Recall}}_{w}$ are defined as above.

These four metrics offer a holistic evaluation of the model’s performance. In particular, the weighted F1-score should be interpreted as a consolidated measure derived from precision and recall rather than as an independent outcome. Together, they not only assess overall prediction correctness but also quantify per-class robustness, ensuring that DualView-LiteNet maintains a consistent classification performance across all maneuver categories even under class imbalance conditions. Overall, this metric formulation ensures that performance comparisons in Section 5 are both interpretable and internally consistent, thereby facilitating concise and reliable quantitative analysis.

## 5. Experiments

This section presents a comprehensive evaluation of the proposed method and several comparative models on the maneuver pattern classification task using the established Maneuver Pattern Library dataset.

### 5.1. Experiment Settings

#### 5.1.1. Dataset

The Maneuver Pattern Library dataset contains a total of 1264 labeled samples, each representing a synchronized pair of top-view and side-view trajectory images generated from simulated flight data. To ensure a fair evaluation, the dataset is randomly divided into training, validation, and testing subsets in a 5:2:3 ratio, with the class distribution maintained across all splits to avoid imbalance. Each sample is annotated with one of five maneuver categories: climb, descent, left turn, right turn, or loiter, which collectively cover the fundamental flight behavior patterns considered in this study.

In addition to the simulated dataset, we incorporate a real-world flight dataset sourced from ADS-B Exchange, a globally recognized cooperative surveillance network that provides open access to real-time and historical Automatic Dependent Surveillance–Broadcast (ADS-B) messages. From this source, we manually curate and annotate five maneuver categories consistent with the simulated dataset, with 100 real trajectory samples per class. The ADS-B dataset is used exclusively for evaluation: models trained on the simulated Maneuver Pattern Library are directly applied to the real-world trajectories in an inference-only manner, without any fine-tuning or retraining. This cross-domain evaluation protocol is designed to assess the generalization capability of simulation-trained models when exposed to real-flight data with naturally occurring noise and operational variability.

#### 5.1.2. DualView-LiteNet Implementation Details

The implementation of DualView-LiteNet follows the architecture introduced in Section 4.2. To ensure stable optimization, all models are trained using the AdamW optimizer with an initial learning rate of $2\times 10^{-4}$, a weight decay of 0.01, and a cosine annealing schedule for a total of 20 epochs. Batch normalization and dropout are applied to prevent overfitting, and early stopping is adopted based on validation performance. The shared-weight design substantially reduces parameter redundancy while maintaining representational alignment across both views, which helps the model to effectively integrate spatial and altitude-related cues. To demonstrate computational efficiency, DualView-LiteNet contains 34,417,349 parameters and requires only 0.247 GFLOPs per forward pass. This lightweight configuration makes DualView-LiteNet suitable for real-time inference under limited computational resources.

#### 5.1.3. Baseline Settings

We compare our proposed method with several baseline models, including a traditional SVM [34] and three dual-view deep learning baselines: CNN, DualView-SelfAttn, and DualView-CrossAttn.

SVM [34] serves as a classical baseline representative of traditional machine learning approaches. Each input sample consists of two image views (top and side), from which handcrafted features are extracted independently. Specifically, we employ a combination of color histograms, Local Binary Pattern (LBP) texture descriptors, and Canny edge features to characterize appearance, texture, and contour information. The extracted features from both views are concatenated into a single representation with a dimensionality of approximately 1588, which is then fed into an SVM [34] classifier. An SVM [34] with a radial basis function (RBF) kernel is adopted. The penalty parameter is set to C=1.4 based on validation performance, and the spread parameter of the RBF kernel is set to γ=0.003. Other kernel parameters, such as degree and coef0, are not used in our configuration. The model is trained using a one-vs-rest strategy for multi-class classification. For completeness, if SIFT and HOG descriptors are used, SIFT produces a set of local descriptors of size (N,128) for each image, and the mean vector is computed to obtain a 128-dimensional representation. HOG features are extracted as a one-dimensional vector and concatenated with the SIFT representation before being fed into the SVM [34] classifier.

As a deep learning baseline, we implement a CNN based on a conventional convolutional neural network [70] with two independent branches for processing the top-view and side-view images separately. Each branch extracts spatial features from its respective input, and the resulting feature maps are concatenated and passed through a fully connected classifier. The network architecture, including convolutional layers, activation functions, and hyperparameters, is kept consistent with our proposed model to ensure a fair comparison. Unlike the proposed Siamese-based design, CNN does not share weights between the two branches, which may limit its ability to enforce feature consistency across views.

To further investigate the effect of attention mechanisms in dual-view modeling, we introduce DualView-SelfAttn as an attention-enhanced baseline. In this model, each view is first processed by a shared CNN backbone, after which multi-head self-attention is applied independently within each view to capture long-range spatial dependencies. The attended features from the two views are then flattened, concatenated, and fed into the same classification head as in CNN. This baseline evaluates whether enhancing intra-view feature representation via self-attention alone is sufficient for improving maneuver recognition.

In addition, we construct DualView-CrossAttn to explicitly model inter-view interactions. Based on the same shared CNN backbone, this model introduces bidirectional cross-attention between the top-view and side-view feature sequences, allowing each view to selectively attend to informative regions of the other. The resulting cross-attended features from both views are concatenated and passed to the classifier. By directly modeling cross-view dependencies, DualView-CrossAttn serves as a strong attention-based baseline to assess the effectiveness of explicit inter-view information exchange compared with the proposed fusion strategy.

### 5.2. Experiment Results

The quantitative evaluation is conducted using four standard metrics: overall accuracy, weighted precision, weighted recall, and the weighted F1-score, providing a comprehensive assessment of classification performance across all maneuver categories. The detailed results are summarized in Table 2.

The SVM [34] baseline attains an overall accuracy of 70.87%, with precision, recall, and F1-score values of 0.74, 0.67, and 0.67, respectively. These results indicate that handcrafted features can partially describe maneuver patterns but struggle to capture complex spatiotemporal characteristics and multi-view correlations. DualView-CrossAttn introduces an explicit cross-attention mechanism to model inter-view feature interactions. While this design improves accuracy to 72.41% compared with the SVM [34] baseline, its precision (0.46) and F1-score (0.51) remain relatively low, suggesting that early-stage cross-view interaction without sufficiently discriminative view-specific representations may introduce noise and limit classification reliability. DualView-SelfAttn applies self-attention within each view branch to enhance intra-view feature modeling. This leads to further improvements, achieving 77.87% accuracy and balanced precision, recall, and F1-score values of 0.62, 0.60, and 0.62, respectively. The results indicate that strengthening view-specific feature representations is beneficial, although the lack of explicit cross-view alignment still constrains overall performance. In contrast, the CNN [70] achieves substantial gains across all metrics, reaching 93.84% accuracy, 0.94 precision, 0.92 recall, and a 0.93 F1-score, demonstrating the effectiveness of end-to-end feature learning for maneuver pattern recognition. However, the use of independent branches limits cross-view consistency, leaving room for further improvement.

The proposed DualView-LiteNet achieves the best overall performance, with 97.64% accuracy and uniformly high scores in precision (0.98), recall (0.98), and F1-score (0.98). This consistent improvement across all evaluation metrics highlights the model’s capability to jointly learn spatial and temporal-related features while maintaining strong cross-view coherence through shared-weight training. Overall, the results confirm that DualView-LiteNet significantly enhances both precision and recall while preserving high classification reliability across maneuver categories, demonstrating its superior robustness and generalization capability on the Maneuver Pattern Library dataset.

**Figure 8:**
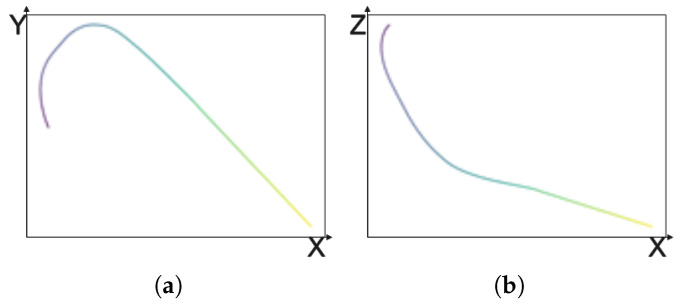
Representative misclassification case. (**a**) Top view; (**b**) side view. A color gradient from dark to light indicates the progression from the start to the end of the trajectory.

Although the proposed DualView-LiteNet achieves consistently high accuracy across maneuver categories, an examination of representative failure cases reveals meaningful insights into the model’s limitations. Misclassifications primarily occur in boundary maneuvers where geometric patterns partially overlap. For example, loiter segments—many of which are generated through data augmentation due to the scarcity of real samples—sometimes present incomplete circular shapes. In such cases, their horizontal-view signatures closely resemble those of left or right turns, both involving sustained heading changes and similar centripetal motion patterns, as illustrated in Figure 8. This overlap can lead the model to incorrectly classify an incomplete loiter maneuver as a turning action. These errors typically arise when the distinguishing features are weak or when the trajectory lies near the class decision boundary, causing ambiguity in the dual-view projections. Such observations suggest that the current feature representation, while effective, still lacks sensitivity to global motion continuity, such as the persistence and completeness of cyclic motion in loiter maneuvers. Future improvements may focus on incorporating duration- or cycle-aware descriptors as well as collecting additional hard samples near class boundaries to strengthen the model’s ability to discriminate between highly similar maneuver types.

### 5.3. Ablation Study

To investigate the functional contribution of each module within DualView-LiteNet, this section presents ablation experiments focusing on five key aspects: the convolutional kernel size, number of convolutional layers, feature fusion strategy, connection layer configuration, and activation function.

#### 5.3.1. Effect of Convolutional Kernel Size on DualView-LiteNet Performance

The convolutional kernel size plays a critical role in determining the receptive field and feature extraction capability of the Siamese network. A larger receptive field allows the model to capture broader spatial context but may lead to the loss of fine-grained details, whereas a smaller kernel emphasizes local information.

In this experiment, kernel sizes of 3×3, 5×5, and 7×7 were compared under identical settings: ReLU activation, batch size of four, two convolutional layers, feature fusion by concatenation, and a fully connected connection layer.

As shown in Table 3, the 3×3 kernel performs overwhelmingly better in this setting. This result indicates that the network is highly sensitive to receptive field size when processing dual-view inputs. In our trajectory library, maneuver patterns are dominated by line-shaped and fine-grained structures. Using a 5×5 kernel causes the model to overfit, achieving low test accuracy despite converging on the training set. With a 7×7 kernel, the receptive field becomes too large, leading to the poor extraction of local trajectory details; as a result, the loss fails to converge and overall accuracy remains near random chance. Consequently, the 3×3 convolutional kernel offers the optimal balance, preserving essential local features while maintaining stable and effective training.

#### 5.3.2. Effect of the Number of Convolutional Layers on DualView-LiteNet Performance

The convolutional branches in the Siamese network are responsible for learning spatial and structural information from the input data. The number of convolutional layers directly influences the model’s representation capacity and training stability. Too many layers may cause overfitting or convergence difficulties, while too few layers may lead to insufficient feature extraction.

To identify the optimal configuration, we tested models with twor, three, and four convolutional layers under fixed settings: ReLU activation, 3×3 kernels, a batch size of four, concatenation fusion, and a fully connected connection layer.

As indicated in Table 4, the configuration with two convolutional layers performs best for this dataset. This result suggests that the maneuver patterns in the Maneuver Pattern Library contain distinctive yet relatively local geometric structures, which can be effectively captured by a shallow convolutional stack. In contrast, adding more layers introduces excessive nonlinearity and parameterization, which destabilizes training and leads to severe performance degradation. Although the four-layer configuration partially recovers accuracy, it still shows reduced generalization capability, indicating that deeper architectures may overfit or extract redundant transformations that are unnecessary for this domain. Overall, the two-layer design provides the best trade-off between representation capacity, optimization stability, and generalization, which aligns with the lightweight design philosophy of DualView-LiteNet.

#### 5.3.3. Effect of Feature Fusion Strategies on DualView-LiteNet Performance

For dual-view maneuver pattern classification, an effective feature fusion strategy is essential to integrate complementary information from both perspectives. Three fusion strategies were compared: (1) concatenation, which connects feature vectors along the feature dimension; (2) addition, which performs element-wise summation with equal weighting (1:1); and (3) multiplication, which performs element-wise products to emphasize inter-view interactions. The settings were identical to previous experiments (ReLU, 3×3 kernels, a batch size of four, two convolutional layers, and fully connected layer).

According to Table 5, all three fusion strategies perform reasonably well. However, concatenation yields the highest accuracy and most balanced performance across all metrics. This advantage can be attributed to its ability to retain complete feature representations from both views, allowing the subsequent fully connected layers to autonomously learn optimal cross-view relationships. In contrast, addition imposes a rigid 1:1 weighting scheme that assumes equal contribution from the two views—an assumption that may not hold for trajectory data where top-view and side-view cues have different discriminative strengths. The element-wise summation also risks mutual cancellation when feature signs differ, leading to the loss of complementary information. Multiplication further amplifies this issue: while it highlights co-occurring feature activations, it suppresses mismatched or low-magnitude responses, causing overly sparse representations that may hinder downstream classification. These observations indicate that concatenation provides the most flexible and expressive fusion mechanism, enabling DualView-LiteNet to fully exploit cross-view complementarity without imposing restrictive structural assumptions.

#### 5.3.4. Effect of Connection Schemes on DualView-LiteNet Performance

In classification tasks, the extracted features must be projected into the target label space. This projection can be implemented either via a fully connected layer (composed of multiple linear transformations and nonlinear activations) or a single linear transformation without hidden layers. This experiment aims to evaluate whether the use of a fully connected structure is necessary for mapping fused features to maneuver categories. The settings were identical to prior experiments (ReLU, 3×3 kernels, a batch size of four, two convolutional layers, and concatenation fusion).

As shown in Table 6, the linear mapping scheme performs significantly worse than the fully connected configuration. A single linear transformation imposes a strict linear separability assumption on the fused feature space, which is insufficient for modeling the complex, nonlinear interactions between dual-view representations—particularly for maneuver patterns characterized by subtle geometric variations. In contrast, introducing a fully connected structure enriches the model’s expressive power by stacking multiple linear transformations with nonlinear activations, enabling the network to learn hierarchical decision boundaries rather than relying on a single global projection. Furthermore, the fully connected layers help to re-balance the contributions from the two views after concatenation, implicitly performing feature reweighting and cross-view coupling that a linear layer cannot achieve. These advantages collectively explain the substantial performance gap, demonstrating that nonlinear connection schemes are essential for achieving robust and high-fidelity maneuver pattern discrimination in DualView-LiteNet.

#### 5.3.5. Effect of Activation Functions on DualView-LiteNet Performance

The activation function plays a crucial role in introducing nonlinearity to the network, directly influencing its representational power. To compare the effects of different activation functions, we tested Sigmoid, Tanh, and ReLU under consistent experimental settings.

As shown in Table 7, it is evident that both Sigmoid and Tanh suffer from gradient vanishing issues during training, resulting in poor classification accuracy and limited generalization ability. Beyond the vanishing gradient problem, these bounded activation functions also compress feature values into narrow numeric ranges, which reduces feature discriminability, which is particularly detrimental for maneuver patterns where subtle local variations must be preserved across dual views. Additionally, Sigmoid and Tanh introduce higher computational costs and a slower convergence due to their expensive exponential operations. In contrast, ReLU not only avoids saturation but also promotes sparse feature activation, enabling the network to emphasize salient geometric cues while suppressing uninformative background patterns. This sparsity-driven selectivity is especially beneficial for the Maneuver Pattern Library, where fine-grained trajectory structures dominate. Therefore, ReLU provides a better trade-off between nonlinearity, optimization efficiency, and feature expressiveness, making it the most suitable activation function for the DualView-LiteNet architecture.

#### 5.3.6. Effect of Data Augmentation on DualView-LiteNet Performance

To evaluate the effectiveness and physical plausibility of the proposed data augmentation strategies, we conduct an ablation experiment comparing DualView-LiteNet trained with and without augmentation. The augmentations include (i) spatial translation within small local offsets to simulate realistic GPS drift or sensor localization noise, and (ii) temporal subsampling to mimic variations in sampling frequency commonly observed in airborne sensing platforms.

Table 8 summarizes the results. Without augmentation, the model achieves an accuracy of 0.9251, a recall of 0.91, a precision of 0.92, and an F1-score of 0.92. After applying both spatial and temporal augmentations, performance improves substantially to an accuracy of 0.9764, a recall of 0.98, a precision of 0.98, and an F1-score of 0.98. Beyond the numerical improvement, a deeper inspection reveals that augmentation effectively mitigates overfitting by preventing the network from memorizing the limited geometric variations present in the Maneuver Pattern Library. The spatial perturbations introduce realistic deviations consistent with sensor noise, enabling the model to develop invariance to small trajectory shifts. Meanwhile, temporal subsampling exposes the network to diverse motion rhythms, improving robustness against varying sampling frequencies and trajectory pacing. These combined effects enrich the data distribution in a physically meaningful manner, strengthening the model’s ability to generalize to unseen trajectories while preserving the intrinsic structure of each maneuver pattern.

### 5.4. Real-World Evaluation on ADS-B Dataset

To further assess the practical relevance of the proposed approach, we evaluate all models on a real-world flight dataset sourced from ADS-B Exchange, following an inference-only protocol. All models are trained exclusively on the simulated Maneuver Pattern Library and directly applied to the real-world trajectories without any fine-tuning or retraining. This setting reflects a strict cross-domain evaluation scenario and is designed to examine whether simulation-trained models can generalize to real-flight data with naturally occurring noise and operational variability. As shown in Table 9, all methods exhibit a noticeable performance drop when transferred from simulated data to real-world ADS-B trajectories, which is expected due to differences in data distributions, sensing noise, and maneuver execution characteristics. Nevertheless, the relative performance ranking among different models remains consistent across both datasets. Traditional SVM [34] struggles to generalize to real-world data, achieving only 20.0% accuracy, indicating the limited transferability of handcrafted features. Attention-based baselines, including DualView-SelfAttn and DualView-CrossAttn, demonstrate improved robustness compared to SVM [34], but their performance is still constrained by the insufficient modeling of cross-view feature consistency. In contrast, the proposed DualView-LiteNet achieves the best performance on the real-world dataset, reaching 65.0% accuracy and an F1-score of 0.64. Despite the absence of real-data supervision during training, the proposed model consistently outperforms all baselines, suggesting that the shared-weight dual-view design effectively captures maneuver-discriminative patterns that are less sensitive to domain shifts. These results provide empirical evidence that simulation-based maneuver primitive learning can offer meaningful generalization to real-flight data, thereby partially addressing concerns regarding the practical validity of simulation-driven approaches. Overall, this real-world inference evaluation highlights the practical robustness of the proposed framework and underscores the effectiveness of dual-view shared representation learning in mitigating sim-to-real performance degradation.

## 6. Conclusions

This study presented DualView-LiteNet, a lightweight dual-view temporal classification framework designed to extract and fuse complementary spatial–temporal cues from synchronized top-view and side-view trajectory sequences. To provide a controlled environment for validating the core capability of the proposed architecture, we constructed a simplified maneuver dataset composed of several standard, single-type maneuvers. This dataset serves primarily as a proof-of-concept platform rather than a final application-oriented benchmark. The experimental results show that DualView-LiteNet effectively captures both horizontal and vertical motion characteristics, consistently outperforming traditional SVM-based methods, dual-view attention-based baselines, and conventional CNN architectures on the simulated dataset. The comparative analysis indicates that introducing explicit cross-view interaction mechanisms improves performance over handcrafted feature and simple fusion baselines, while shared-weight dual-view convolutional learning further strengthens feature consistency and discriminative capability. While a strong performance on idealized data alone does not guarantee its direct applicability to real-world flight scenarios, additional inference experiments on real-world ADS-B trajectories demonstrate that the model trained purely on simulated data can generalize to realistic flight patterns to a certain extent. This observation supports the validity of the proposed simulation-driven formulation and suggests that the learned dual-view representations capture maneuver-related structures that are not limited to the synthetic domain.

Accordingly, this work should be regarded as an initial step toward more comprehensive maneuver understanding rather than a complete end-to-end solution. The proposed dual-view framework is model centric and general in its design; its ability to align multi-view temporal features is expected to remain beneficial when extended to more complex, noisy, and compound maneuver patterns. Nevertheless, substantial future efforts are still required to systematically evaluate robustness under more diverse and less controlled conditions.

Future work will prioritize (i) a deeper investigation of sim-to-real generalization using larger and more diverse real-world flight datasets, (ii) expanding the maneuver library to include compound, non-ideal, and mixed maneuvers that better reflect practical flight behaviors, and (iii) enhancing DualView-LiteNet with more expressive temporal modeling mechanisms, additional sensing cues, and domain adaptation strategies. These directions are essential for further improving the robustness, interpretability, and practical value of multi-view maneuver pattern classification.

## Figures and Tables

**Table 1:** Aircraft parameters.

Length	Wingspan	Height	Maximum Flight Speed	Maximum Takeoff Weight	Service Ceiling	Combat Radius
(m)	(m)	(m)	(Mach)	(kg)	(m)	(km)
19.1	19.54	4.88	2.34	33,724	15,200	926

**Table 2:** Maneuver pattern classification benchmark on Maneuver Pattern Library. The upward arrows (↑) indicate that higher values represent better performance.

Method	Simulated Dataset				Real-World Dataset			
	Accuracy↑	Precision↑	Recall↑	F1-Score↑	Accuracy↑	Precision↑	Recall↑	F1-Score↑
SVM [34]	0.7087	0.74	0.67	0.67	0.2000	0.04	0.20	0.07
DualView-CrossAttn	0.7241	0.46	0.58	0.51	0.3120	0.30	0.31	0.23
DualView-SelfAttn	0.7787	0.62	0.60	0.62	0.4080	0.45	0.41	0.40
CNN [70]	0.9384	0.94	0.92	0.93	0.602	0.58	0.60	0.58
DualView-LiteNet (Ours)	0.9764	0.98	0.98	0.98	0.6500	0.66	0.65	0.64

**Table 3:** Performance comparison across different convolutional kernel sizes. The upward arrows (↑) indicate that higher values represent better performance.

Convolutional Kernel Size	Accuracy↑	Precision↑	Recall↑	F1-Score↑
3×3	0.9764	0.98	0.98	0.98
5×5	0.2350	0.18	0.12	0.14
7×7	0.2342	0.05	0.23	0.09

**Table 4:** Performance comparison across different number of convolutional layers. The upward arrows (↑) indicate that higher values represent better performance.

Number of Convolutional Layers	Accuracy↑	Precision↑	Recall↑	F1-Score↑
2	0.9764	0.98	0.98	0.98
3	0.4098	0.71	0.41	0.38
4	0.9426	0.94	0.93	0.93

**Table 5:** Performance comparison across different fusion methods. The upward arrows (↑) indicate that higher values represent better performance.

Fusion Method	Accuracy↑	Precision↑	Recall↑	F1-Score↑
Concat	0.9764	0.98	0.98	0.98
Add	0.9426	0.94	0.94	0.94
Mul	0.9099	0.92	0.91	0.91

**Table 6:** Performance comparison across different connection schemes. The upward arrows (↑) indicate that higher values represent better performance.

Connection Scheme	Accuracy↑	Precision↑	Recall↑	F1-Score↑
Fully Connected	0.9764	0.98	0.98	0.98
Linear Mapping	0.8023	0.81	0.79	0.80

**Table 7:** Performance comparison across different activation functions. The upward arrows (↑) indicate that higher values represent better performance.

Activation Function	Accuracy↑	Precision↑	Recall↑	F1-Score↑
Relu	0.9764	0.98	0.98	0.98
Sigmoid	0.2442	0.05	0.23	0.09
Tanh	0.2123	0.04	0.21	0.07

**Table 8:** Performance comparison across different data augmentation settings. The upward arrows (↑) indicate that higher values represent better performance, and the checkmark (✓) denotes that the data augmentation strategy is applied.

Augmentation	Accuracy↑	Precision↑	Recall↑	F1-Score↑
	0.9251	0.92	0.91	0.92
✓	0.9764	0.98	0.98	0.98

**Table 9:** Inference-only maneuver classification results on the real-world ADS-B dataset.

Method	Accuracy↑	Precision↑	Recall↑	F1-Score↑
SVM [34]	0.2000	0.04	0.20	0.07
DualView-CrossAttn	0.3120	0.30	0.31	0.23
DualView-SelfAttn	0.4080	0.45	0.41	0.40
CNN [70]	0.6020	0.58	0.60	0.58
DualView-LiteNet (Ours)	0.6500	0.66	0.65	0.64

## Data Availability

The raw data supporting the conclusions of this article will be made available by the authors on request.
